# Vulvar and vaginal atrophy in four European countries: evidence from the European REVIVE Survey

**DOI:** 10.3109/13697137.2015.1107039

**Published:** 2015-11-19

**Authors:** R. E. Nappi, S. Palacios, N. Panay, M. Particco, M. L. Krychman

**Affiliations:** ^a^ Research Center for Reproductive Medicine, Gynecological Endocrinology and Menopause, IRCCS S. Matteo Foundation, Department of Clinical, Surgical, Diagnostic and Paediatric Sciences, University of Pavia, Pavia, Italy; ^b^ Palacios Institute of Women's Health, Madrid, Spain; ^c^ Imperial College London, London, UK; ^d^ Shionogi Italy, Rome, Italy; ^e^ Southern California Center for Sexual Health and Survivorship Medicine Inc., Newport Beach, CA, USA

**Keywords:** Vulvar and vaginal atrophy, postmenopausal women, REVIVE Survey, Europe, sexual relationships, vaginal dryness, dyspareunia, treatment

## Abstract

**Objectives** The aim of the European REVIVE survey was to achieve a better understanding of vulvovaginal atrophy (VVA), a chronic and progressive condition after menopause. We investigated perceptions, experiences and needs in terms of sexual and vaginal health in a sample of European postmenopausal women.

**Methods** An online internet based survey was conducted in Italy, Germany, Spain and the UK with a total surveyed sample of 3768 postmenopausal women (age: 45–75 years).

**Results** The most common VVA symptom was vaginal dryness (70%). VVA has a significant impact on the ability to be intimate (62%), to enjoy sexual intercourse (72%) and to feel sexual spontaneity (66%). Postmenopausal women with VVA are sexually active (51%), but their sexual drive is reduced. Health-care professionals (HCPs) have discussed VVA with postmenopausal women (62%), but they initiated the conversation only in 10% of the cases. The most common treatments for VVA are over-the-counter, non-hormonal, local vaginal products. Thirty-two per cent of postmenopausal women were naïve to any kind of treatment, whereas discussion with the HCP was relevant to be on current treatment (60% of postmenopausal women that discussed VVA with a HCP vs. 23% who did not). The top reasons for poor compliance with vaginal treatments were: not bothersome enough symptoms (18%); vaginal changes not therapeutically reversed (18%); relief from VVA symptoms (17%). Approximately 45% were satisfied with treatment. The most frequent disliked aspects of treatment were the route of administration or the messiness. The fear of hormones was common in postmenopausal women using vaginal prescription products.

**Conclusions** The European REVIVE survey confirmed that VVA symptoms are frequent in postmenopausal women and demonstrates a significant impact on quality of life and sexual life. However, the condition is still under-diagnosed and under-treated, with a high rate of dissatisfaction for actual available treatments in the four European countries surveyed. The discussion of symptoms with HCPs seems the most critical factor for diagnosis and treatment of VVA.

## Introduction

Vulvar and vaginal atrophy (VVA) is a relatively common condition symptomatically affecting approximately 50% of all postmenopausal women. It is a consequence of reduced estrogenization of urogenital and pelvic tissue that results in a loss of vaginal elasticity, dryness, decreased lubrication, with associated irritation, dyspareunia, and urinary symptoms^1–6^. Previous research has shown that the impact of VVA symptomatology in European women is significant, especially in those achieving their last menopause period, and this is a direct consequence of an aging population^7^.

The disparity between the high prevalence and infrequent clinical diagnosis is documented in medical practice despite the considerable negative impact on quality of life^4^^,^ 8. This inaccuracy is thought to be primarily a consequence of patients’ unwillingness and/or embarrassment to report symptoms to their health-care professional (HCP)^9–11^ and also, from a standpoint of the HCP, the difficulty of approaching this sensitive topic during routine consultations^12^. Associated with this under-diagnosis is a chronic and progressive condition that may not be addressed for a considerable period of time, and therefore more likely to undergo disease progression when left untreated^13^.

Currently available therapeutic options consist of topical over-the-counter (OTC) products (including non-hormonal moisturizers and lubricants applied in the vagina), prescribed systemic hormonal treatment (indicated when other menopausal symptomatology is also present), and prescribed local vaginal estrogen treatments (e.g. creams, suppositories, rings, and tablets)^3^. In addition to the difficulty of achieving an early diagnosis, the most important issues that may compromise patients’ adherence and therapeutic efficacy are the concerns, expressed by both women and HCPs, regarding the long-term safety of using estrogen-containing products and the convenience of topical product application^14^^,^ 15.

During the last decade, many cross-cultural studies, predominantly surveys, have been conducted on postmenopausal women to gain new insight into the impact of VVA in current postmenopausal populations from different geographic distributions^8^^,^ 12 ^,^ 16–20. These studies indicated that VVA symptoms have a global negative effect on sexual health, satisfaction, and sexual behavior, despite accounting for confounding factors due to population heterogeneity and absence of objective criteria to document the real medical condition.

Nevertheless, the existence of clear fragmentary information on therapies provided to patients as well as a lack of exploration of women’s knowledge of available treatments for VVA are evident. Some misconceptions, such as the negative perceptions of estrogen therapy, continue to jeopardize the optimization of VVA management and its effective treatment^4^^,^ 21.

The objective of the present REVIVE-EU (REal Women’s VIews of Treatment Options for Menopausal Vaginal ChangEs-Europe) survey was to characterize women’s knowledge of VVA and its symptomatology in relation to menopause in four European countries, to describe the impact of VVA symptoms on women’s daily life, and to understand current European attitudes to HCP interactions. This focus may help to gain both a better understanding of European menopausal women’s perceptions, experiences, and needs in relation to sexual and vaginal health treatments, and to achieve a significant improvement in the efficacy and therapeutic adherence.

## Methods

### Population

The European REVIVE included a cohort of 3768 postmenopausal women with VVA symptoms from four European countries (1000 from Germany, 1000 from Italy, 1000 from the UK, and 768 from Spain). The inclusion criteria were as follows: menopausal (either natural or surgically induced) women, residence in one of the four study countries, aged between 45 and 75 years old, and who have experienced at least one of the vulvar and vaginal atrophy symptoms (dryness, pain with sex, irritation, tenderness, pain with exercise and/or bleeding).

### REVIVE-EU

**Figure 1.**
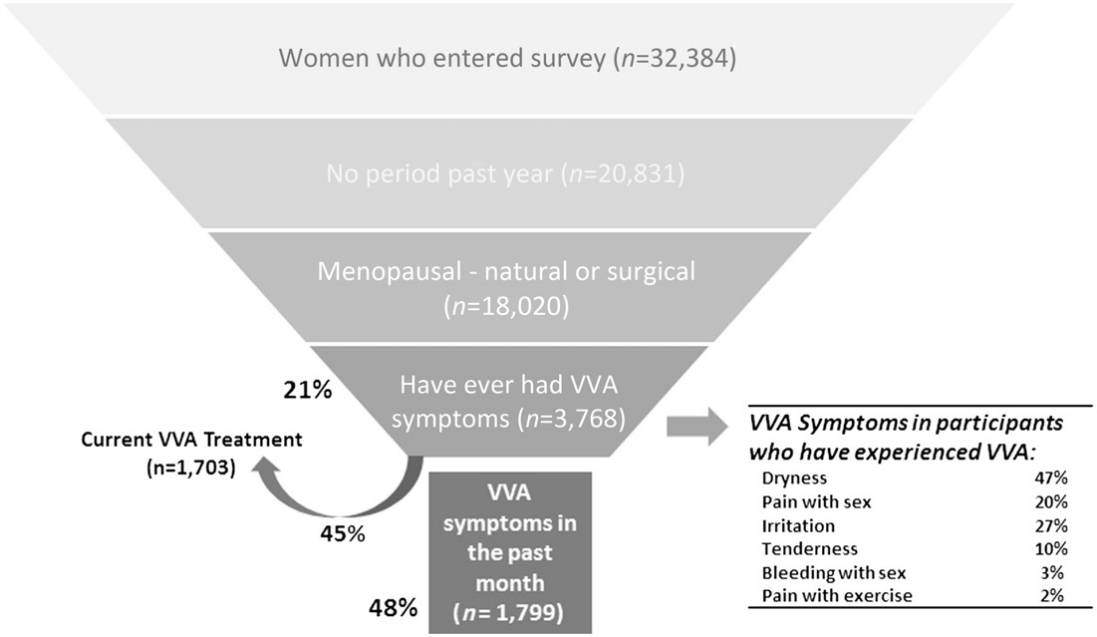
Characteristics of surveyed participants. VVA, vulvovaginal atrophy.

The European REVIVE (REVIVE-EU) survey is a translated and culturally adapted version of the previous USA REVIVE questionnaire (research agency: Eikon Europe; panel used: Toluna Group). The comprehensive online questionnaire was approved by the corresponding accredited institutional review board. The survey participants were appropriately informed of the nature of the study and gave informed consent to participate. Before the beginning of the study period, the online questionnaire was pre-tested on 5% of the UK sample (50 participants) to ensure participants fully understood the survey questions. The last European iteration of the survey lasted 35 min and was designed with a margin error of 3.1% at the 95% confidence interval. The invitation to participate was sent to the target population (postmenopausal women with at least one VVA symptom after the onset of menopause) by the panel (selected by age range), who were compensated with points that can then be redeemed for vouchers or gadgets (but not for products or money). Participants entered the secure online questionnaire portal and completed the survey between June 18, 2014 and July 22, 2014. Prior to the completion of the questionnaire, a three-step screening process was completed (see Figure 1). After the screening, the information collected from the participants included: level of knowledge about VVA and menopausal symptomatology, interactions with HCPs with respect to VVA symptomatology, impact of VVA symptoms on sexual and daily living activities, current or previous use of OTC products for VVA, current or previous use of prescription treatments for VVA, and attitudes toward VVA treatments.

Eligible patients were those who fulfilled all selection criteria and who had valid data for the variable/s considered. There was no imputation of missing data. Descriptive statistics were summarized by relative frequency distributions for categorical variables of the questionnaire.

## Results

### Survey population distribution

The baseline demographics and clinical characteristics of the final sample of 3768 survey participants are summarized in Table 1. For the age demographic, the 50–60-year age range was the most represented range in the sample (60.5%). Among the cohort of participants surveyed (Figure 1), 1799 (47.7%) had experienced VVA symptoms in the past month and 2480 (65.8%) in the past 6 months, respectively. At the moment of starting the survey, 1703 (94.7%) of those with recent VVA symptoms were receiving VVA treatment.

**Table 1.** Baseline characteristics of surveyed population of women reporting symptoms of vulvovaginal atrophy (VVA) (*n* = 3768). Data are given as *n* (%).

*Age* (years)	
45–50	363 (9.6%)
51–55	1163 (30.9%)
56–60	1091 (29.0%)
61–65	715 (19.0%)
66–70	329 (8.7%)
71–75	107 (2.8%)
*Marital status*	
Married	2364 (62.7%)
Divorced	516 (12.9%)
Domestic partnership	349 (8.7%)
Single	283 (7.1%)
Widowed	209 (5.2%)
Separated	128 (3.2%)
*Education/employment*	
Employed	1317 (35.0%)
University education or higher^a^	1380 (36.6%)
*Number of children*	
None	758 (20.1%)
One	910 (24.2%)
Two	1441 (38.2%)
Three	475 (12.6%)
Four or more	184 (4.9%)
Children living at home	1421 (47.2%)
*Prior treatment for VVA symptoms*	2554 (67.8%)
OTC product	1545 (41.0%)
Prescription medication	382 (10.1%)
Prescription and OTC in combination	450 (11.9%)
*Current treatment for VVA symptoms*	1703 (45.2%)
OTC product	1138 (30.2%)
Prescription medication	341 (9.0%)
Prescription and OTC in combination	72 (1.9%)

^a^Includes trade training, degrees and master’s degrees.

OTC, over the counter.

### VVA knowledge and menopause awareness

According to this cohort of postmenopausal European women, 39.9% of the participants were able to directly attribute VVA to the menopausal transition. By contrast, only 8.1% of participants responded that they were not aware of the cause of their VVA symptoms. Additionally, almost 47% of participants were not aware of the VVA condition. Within the cohort of participants that were aware of the VVA condition, this knowledge and information came through direct discussions with their HCP (44.0%), active internet searching (11.0%) or newspaper/journal paper reading (10.0%). In the total sample included, only 16.4% of the participants had been officially clinically diagnosed with VVA.

### VVA symptoms and impact on sexual life and other activities

**Figure 2.**
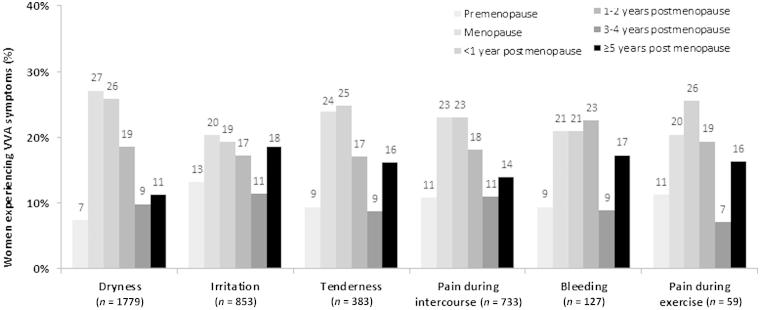
Onset of vulvovaginal atrophy (VVA) symptoms in participants currently suffering from VVA.

The most common VVA-associated symptoms in European postmenopausal women were vaginal dryness (70.0%), followed by vaginal irritation (32.7%), pain during intercourse (dyspareunia) (29.0%) and vaginal tenderness (14.3%). The retrospective report of the onset of symptoms associated or linked with VVA is presented in Figure 2.

Most participants (65% or more) reported the onset of VVA symptoms during the postmenopausal period. The onset of symptoms varied considerably across menopause stages; vaginal irritation was the most likely symptom to occur before menopause (13.1%), while vaginal dryness, pain with exercise and tenderness were the most likely symptoms to begin within the first year after menstrual cessation (25.8%, 25.5% and 24.7%, respectively). The onset of pain with intercourse could occur across all the menopause and postmenopause period, but appears most commonly at the exact time of menopause (23.0%) or the subsequent year (23.0%).

**Figure 3.**
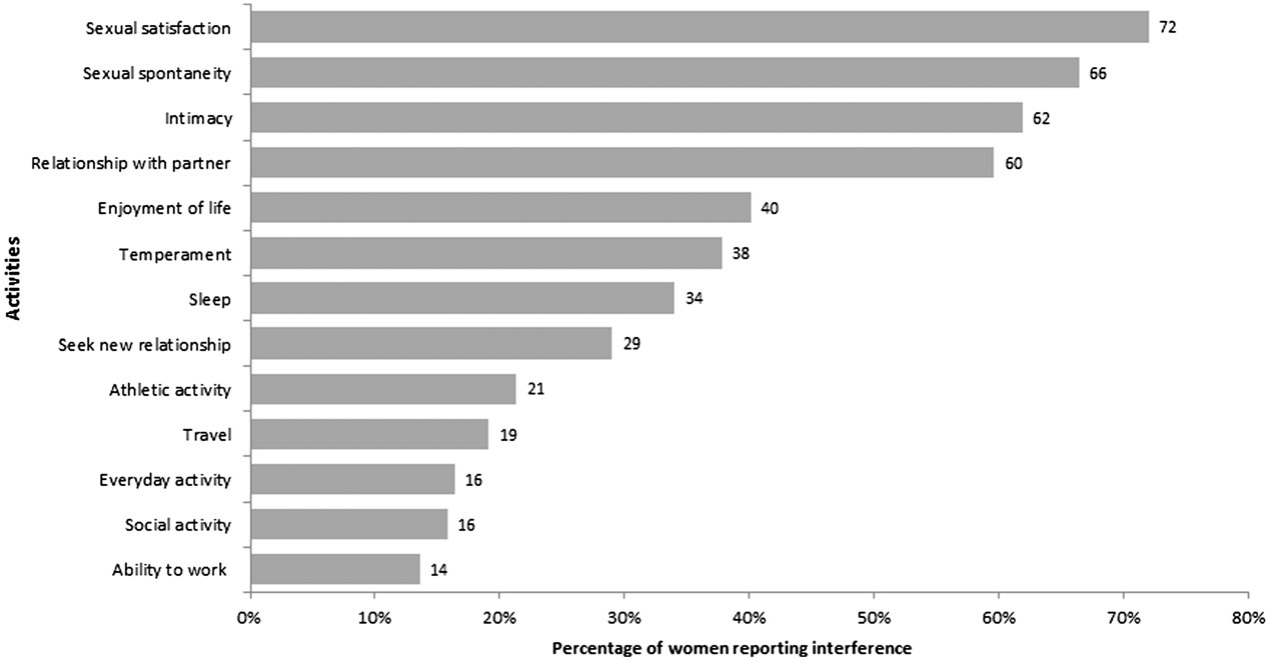
Interference of symptoms of vulvovaginal atrophy with sexual life and other activities.

Overall, VVA symptoms were reported to have the most impact on participants’ sexual satisfaction (72.0% somewhat or very interfering), followed by interferences in sexual spontaneity (66.4%), intimacy (61.9%), and relationship with their partner (59.6%), respectively (Figure 3). About 60% of the participants stated that symptoms were the same or worse now than when they first appeared, especially with regard to pain during intercourse (76.7%). All VVA symptoms were rated as quite bothersome, but pain and bleeding associated with sex rated as the most bothersome symptoms (51.8% and 45.0% of participants rated them as extremely bothersome). Participants also reported that VVA symptoms made them feel uncomfortable (47.7%), 'old' (39.9%), frustrated (17.6%) and/or unattractive (14.4%). When asked about what is the most concerning facet as a result of VVA symptoms, most participants felt losing their sexual intimacy (40.3%) and their youth (19.1%) were the most significant points of concern.

Participants with a partner (77.9%) were co-habitating in 84.5% of cases, 25.5% of which were without sexual activity in the last year. For participants who had sexual activity during the last year, the most important VVA symptoms were described as pain associated with intercourse (82.6%), vaginal and/or vulvar dryness (78.3%) and pelvic tenderness (67.1%). Overall, the survey shows that sexual drive was diminished in about 50% of patients as a direct result of VVA symptomatology.

### Health-care professional relationship

Altogether, 88.8% of participants reported visiting a HCP for their gynecological needs; over half of these HCPs were female physicians (58.4%). Specifically, 2540 participants (67.4%) reported having a gynecologist/obstetrician for their main gynecological needs and 799 (21.2%) had a GP/family practitioner. Overall, 74.1% of the participants consulted their main HCP during the last 12 months. Among participants who reported having a HCP for their gynecological needs, only 10.5% indicated that their HCP usually asked about that participant’s sexual activity during the routine physical examination. By contrast, 65.1% of participants acknowledged that they expect their HCP to initiate a conversation to inquire about menopause-related symptoms.

More than six out of ten participants in the survey (61.6%) had specifically discussed VVA symptoms with their HCP; however, only 10.3% of them said that the HCPs themselves had initiated the conversation. The most commonly reported reasons for avoiding a discussion with their HCP about VVA were beliefs that symptoms were a natural component of the aging process (42.6%), followed by the statement that symptoms were not bothersome enough to be considered problematic and did not warrant discussion with their HCP (26.0%). More than half of the participants (54.6%) had spoken to their partner about VVA symptomatology. Remarkably, depending on the specific symptom, between 36.0% (vaginal/vulvar irritation) and 50.0% (pain associated with sexual intercourse) of participants suffered VVA for more than 6 months before they initiated a discussion with a HCP. Satisfaction with how the HCP handled the first discussion about VVA symptoms was declared in 62.5% of cases.

### Participants' experiences regarding VVA treatments

**Figure 4.**
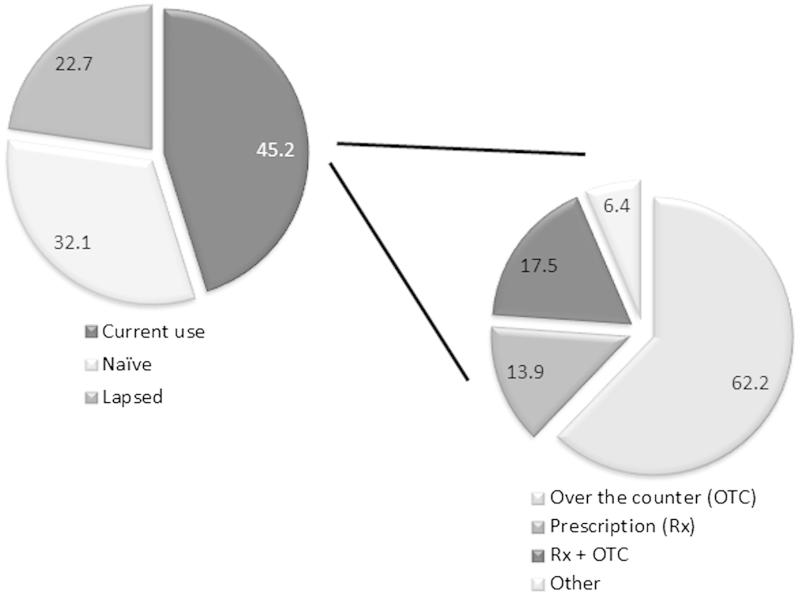
Current use of treatment by participants.

In the total European REVIVE survey, 1703 participants (45.2%) were currently using a VVA-specific treatment (OTC lubricants or moisturizers, prescribed local estrogens, other OTC medications, or herbal therapies), 32.1% were treatment-naïve and 22.7% had lapsed from their treatment schedule at the time of the survey (Figure 4). Women who discussed VVA with a HCP were twice as likely to be current medication users (59.7% vs. 22.7% for those who had not discussed VVA). Within the group of participants under current treatment, 62.2% were using OTC products, compared to 13.9% using vaginal prescription therapies and 17.5% using both kinds of products. These participants initiated treatment for their VVA symptoms in different manners: 17.2% started treatment through a HCP prescription and the recommendation of an OTC product to be used together, another 16.0% began treatment through a HCP prescription without a OTC product recommendation, and another 15.8% started using an OTC product before talking to a HCP or before receiving a specific prescription.

An overall ratio of 39.2% of participants abandoned their treatment at some point, and justified it mainly because they did not consider their symptoms bothersome enough to continue treatment (18.4%), the medication was not able to reverse the vaginal changes (18.3%), the consolidated relief from vaginal/vulvar symptoms (17.5%), the price and high expense of the treatment (10.4%), and their assumption that symptoms would diminish with time (10.4%). In patients who completed their OTC treatment or who were currently taking it, 45.2% reported overall satisfaction; while 8.4% continued to be dissatisfied (46.4% had a neutral opinion). By contrast, in patients who completed their prescription medication or who were currently taking it, 44.3% showed a global satisfaction with it, while only 10.4% were dissatisfied (45.3% neutral).

**Figure 5.**
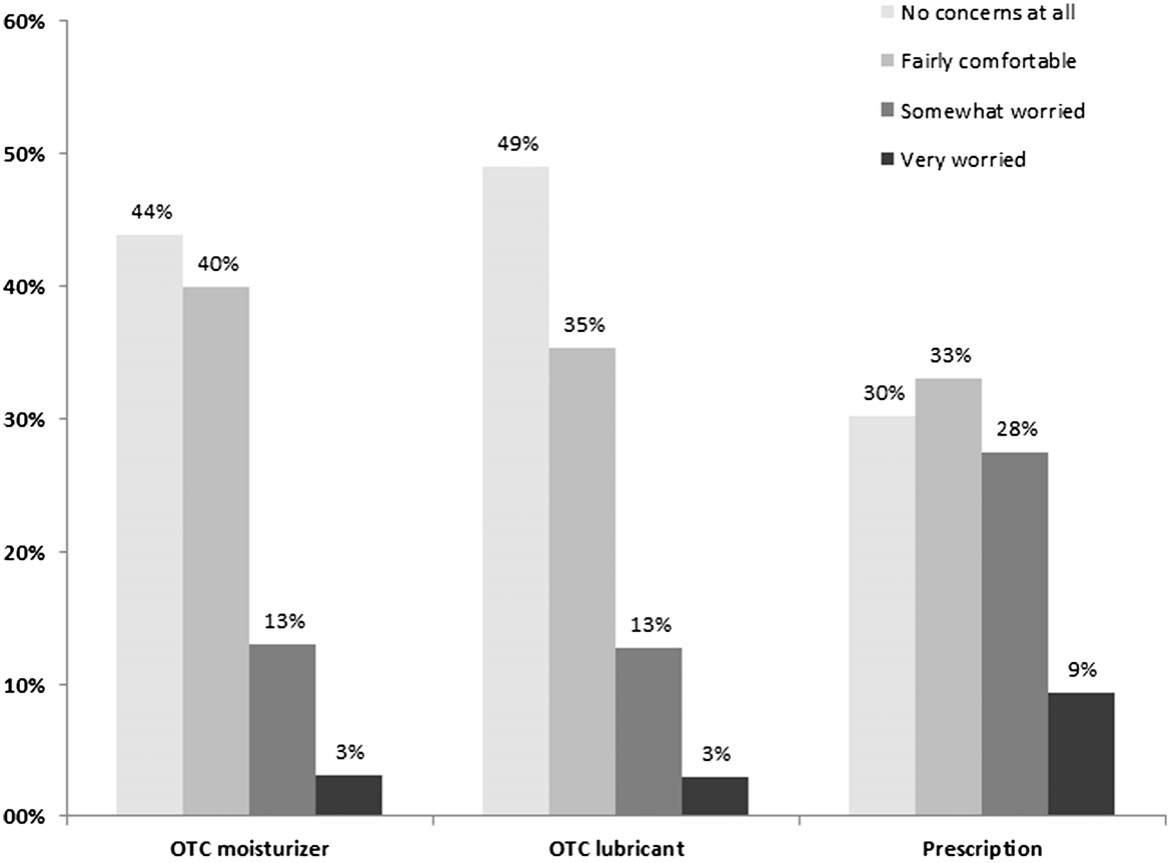
Proportion of participants concerned with long-term use of their current vulvovaginal atrophy medication. OTC, over the counter.

The percentages of participants who were worried or concerned about the long-term use of VVA medication are summarized in Figure 5. Participants using vaginal prescription products showed a higher degree of concern with long-term use and felt themselves less comfortable than those with OTC moisturizers or lubricants. The main worries expressed by the participants about the long-term use and safety of VVA medications were the concerns about side-effects (55.7%), followed by aspects related to hormone exposure and a possible predisposition for developing cancer (24.6%). The concerns about the unknown implications of long-term use (19.7%) were also mentioned by participants. Within the group of participants who experienced symptoms, but did not use medication, 5.0% claimed significant concerns/worry about breast cancer. On the contrary, in participants currently using treatment, breast cancer concerns tended to be lower (5.1% in those with prescription, 2.4% in those using an OTC moisturizer, and only 1.0% in those using OTC lubricants).

Survey participants also expressed their views on VVA treatment difficulties (Table 2). Users of OTC moisturizers were most worried about efficacy issues, such as the inability to restore the vagina to its natural state (27.8%) or the limitation on symptom relief (13.1%). They also had concerns related to the price of the product (14.2%) and its impact on sexual spontaneity (15.0%). Participants who used OTC vaginal lubricants were mainly worried by the limitations of the product in restoring the vagina to its natural state (28.2%) and the administration of the products, which were considered messy (18.5%). Almost 10% of these users also considered the product to be expensive. In terms of administration, 54.0% of participants who have a priority with route of administration showed a clear preference for an oral pill, whereas only 46.0% preferred or somewhat preferred a vaginally-administered product for the treatment of VVA problems. Participants who were taking prescription vaginal products for VVA symptoms had lower but still significant issues with the ability of the product to restore the natural state of the vagina (25.7%). They were also worried with safety issues like: long-term effect on safety (21.3%), hormone exposure (16.5%), vaginal discharge (15.0%), other side-effects (16.3%), as well as the possibility of estrogen absorption by their partner (3.9%).

**Table 2.** Views of therapies for vulvovaginal atrophy (VVA) in participants currently using treatment. Data are given as *n* (%).

	OTC personal vaginal moisturizer (*n* = 809)	OTC vaginal lubricant (*n* =308)	Prescription vaginal (*n* =381)
*Issues related to route of administration*			
Messy	76 (9.4%)	57 (18.5%)	51 (13.3%)
Not discrete	28 (3.5%)	8 (2.6%)	12 (3.1%)
Not an oral pill	68 (8.4%)	14 (4.5%)	28 (7.3%)
Do not like touching body	17 (2.1%)	7 (2.3%)	8 (2.1%)
*Issues related to convenience*			
Interrupts my daily activities/life	12 (1.5%)	4 (1.3%)	6 (1.6%)
Inconvenient to administer	64 (7.9%)	22 (7.1%)	58 (15.2%)
Cannot be sexually spontaneous	121 (15.0%)	39 (12.7%)	31 (8.1%)
Difficult dosing schedule	28 (3.5%)	4 (1.3%)	17 (4.5%)
Procedure of administering treatment	52 (6.4%)	13 (4.2%)	44 (11.5%)
*Issues related to side-effects/safety*			
Concern about breast cancer	31 (3.8%)	4 (1.3%)	43 (11.3%)
Concern about hormone exposure	41 (5.1%)	5 (1.6%)	63 (16.5%)
Concern about safety in long-term use	88 (10.9%)	36 (11.7%)	81 (21.3%)
Vaginal discharge	51 (6.3%)	16 (5.2%)	57 (15.0%)
Concern about other side-effects	70 (8.7%)	16 (5.2%)	62 (16.3%)
Experienced side-effects	14 (1.7%)	4 (1.3%)	6 (1.6%)
Partner absorbing estrogen	14 (1.7%)	2 (0.6%)	15 (3.9%)
*Issues related to efficacy*			
Vagina not restored to natural state	225 (27.8%)	87 (28.2%)	98 (25.7%)
Not enough relief of symptoms	106 (13.1%)	37 (12.0%)	49 (12.9%)
Takes a long time to start working	61 (7.5%)	18 (5.8%)	46 (12.1%)
*Other*			
Expensive	115 (14.2%)	30 (9.7%)	26 (6.8%)
Negative impact on intimacy	23 (2.8%)	7 (2.3%)	3 (0.8%)

OTC, over the counter.

## Discussion

The European REVIVE was conducted in the largest cohort of postmenopausal women included in a survey of this nature to date. The survey of 3768 postmenopausal women with diagnosed VVA symptomatology included in the European version of the REVIVE study highlights the persistent and significant lack of awareness of the underlying pathology and its origin. Furthermore, this study has detected several limitations in clinical and therapeutic management of this chronic condition in the four European counties surveyed. For example, participants’ main concerns about VVA symptoms were centered on interference with sexual behavior and loss of intimacy. These results follow a similar pattern to those observed previously (i.e. in the VIVA Survey, 64% had sexual intimacy interference and 32% had interference with the loving relationship with a partner)^8^^,^ 12 ^,^ 17 ^,^ 18 ^,^ 21. This study clearly shows the important impact of VVA symptoms in European women after menopause, especially in aspects related to sexual satisfaction and spontaneity, with the ability to be intimate, and the ability to establish a trusted relationship with partners.

Almost four out of ten European participants with VVA symptoms were able to associate the symptoms with menopause as a causal factor, which implies a higher identification of VVA as a chronic menopause-linked syndrome than in previously published surveys^8^^,^ 9 ^,^ 20. This difference could be, in part, related to a difference in the ability to access the resources necessary for a VVA diagnosis and differences between medical evaluation methodologies in Europe and in the United States. Additionally, it should be noted that the nomenclature of VVA has recently evolved to be part of the more extensive genitourinary syndrome of menopause (GSM) according to the recommendations of the International Vulvovaginal Atrophy Terminology Consensus Conference Panel, as a more accurate and all-encompassing term in order to improve and ease conversations between patients and their HCPs^22^.

Although the majority of participants experienced one unique symptom associated with VVA, a very high proportion of patients experienced two or more symptoms before consultation with a HCP. This pattern is related with another observation concerning the waiting time before discuss their problems: between 36% and 50% of participants (depending on the observed symptoms) waited more than 6 months to discuss them with a HCP. This confirms that VVA is an under-recognized, under-diagnosed and undertreated condition among postmenopausal participants who, although experiencing bothersome symptomatology, quite often do not seek medical help^8^^,^ 9 ^,^ 23 ^,^ 24. Vaginal dryness ranked first in the top symptoms to appear in postmenopause, being also the symptom which most participants clearly identified as being directly related to the menopause process^25^.

Symptoms were usually reported as not being resolved over time or in fact worsening as time progressed, causing discomfort and impacting sexuality and the quality of life, even for participants who reported satisfaction with treatments. Pain during intercourse was the most bothersome symptom and was associated with a significant impact on healthy and satisfying intimate relations^26^, which are a desired effect of the treatment stated by the surveyed participants. The study shows an unmet medical need which is related to poor public awareness and lack of education on VVA symptoms, terminology and the impact of the disease on the woman and her partner. Although more participants are able to associate their VVA symptomatology with menopause than previously reported^20^, the majority are still unaware that the symptoms are due to a recognized and treatable medical condition, and that they are strongly compromising their ability to maintain an active and healthy level of sexual intimacy^27^. Educational and public awareness campaigns are warranted.

Even though the discussion of symptoms with a HCP has a significant impact on the incidence of the diagnosis of VVA, an important conclusion of our study is the proven difficulty and inadequacy of fluent communication related to VVA symptoms between patients and HCPs. First, the majority of participants wait several months or years with active symptoms before speaking to a HCP and only 10% of HCPs initiate a discussion on VVA symptoms. Second, nearly 40% of participants had never discussed their symptoms with a HCP. Finally, only 16% of symptomatic participants received a formal VVA diagnosis by their HCP. These observations demonstrate the importance of an increased awareness and responsiveness of HCPs regarding VVA and its impact in postmenopausal women. A paucity of knowledge related to available treatments remains, although differential behaviors could be identified between primary-care clinicians and the obstetrics/gynecology specialists, with the latter group being more likely to routinely assess patients’ sexual activities (63%) and sexual problems (40%)^8^^,^ 9 ^,^ 28 ^,^ 29.

Within the main findings of the current European survey, one must highlight the low satisfaction and significant concerns expressed by symptomatic participants with VVA treatment (OTC and/or prescription medication), mainly justified by a skeptical view that available treatments can effectively reverse the vaginal changes and by safety and convenience limitations. The incidence of the OTC/prescription combination is low. Remarkably, one in three participants remained untreated, notwithstanding the fact that lubricants should be used as the first-line treatment to ease sexual intercourse according to the North American Menopause Society guidelines^30–32^. In addition, 25% of treated participants discontinued their treatment, and close to 40% abandoned it completely. As a consequence, the role of HCPs appears important in two main therapeutic aspects: (1) the HCP must be more active in creating diverse educational awareness on how to deal with VVA symptoms; and (2) the HCP must facilitate discussions with their patients on menopause symptomatology and therapeutic needs associated with sexual health. Lastly, even if concerns regarding VVA prescription treatments, usually associated with safety issues of the estrogen therapy, are expressed by participants, the HCP must be well versed in the etiology of the condition (decrease in endogenous estrogen after the menopause) and that the benefits and efficacy of minimally absorbed local vaginal estrogen therapy in alleviating VVA symptoms have been extensively proven^31^^,^ 33. Patient education and support may help in patient compliance with treatment. Furthermore, HCPs must be aware of the risk–benefit profile of the products that are available for the treatment of VVA and possible concerns of the patient should be addressed.

Some limitations of this survey are a consequence of the characteristics and nature of surveys. The data of the European REVIVE study came from a self-reported, on-line questionnaire, a type of questionnaire that suffers from a certain recall bias effect and is subject to respondent bias through the limitations of reporting subjective symptoms from an objective condition. Another limitation may be attributable to sociocultural and country-specific differences present in Europe. For example, participants were difficult to detect, the level of information that could be acquired was inconsistent, open-mindedness to discuss VVA symptomatology was variable, and therapeutic products differed in availability, amongst others. Cross-cultural variables were not addressed. Nevertheless, the sample included in the REVIVE study represents the largest cohort of European postmenopausal women interviewed on VVA symptomatology; therefore this may represent and reflect a 'real-world' perspective on the VVA condition and its therapeutic opportunities. The results may, in fact, be generalizable to the European population as a whole due to the diversity of the patients surveyed.

## Conclusions

The European REVIVE study has highlighted the significant impact of VVA symptomatology in women living in four European countries. Direct HCP–patient communication and interaction still suffers from a considerable lack of dialogue that must be actively promoted from the professional point of view in order to convey updated and complete information about the VVA etiology and its progressive nature to patients. Proactivity by the HCP should be centered on educating the patients with updated information on VVA, its diagnosis, assessment and the risks/benefits of a suitable therapeutic approach.

The European REVIVE study leads to the conclusion that a well-developed educational program focusing on these issues will have a positive impact on the adherence to treatment and the overall therapeutic efficacy with direct impact on satisfaction and quality of life of European women suffering from VVA.
